# Molecular Effects of *Parkia speciosa* Hassk. Empty Pod Extract in Colon Cancer: A Transcriptomic and Proteomic Perspective

**DOI:** 10.3390/ijms27125606

**Published:** 2026-06-21

**Authors:** Athit Chaiwichien, Supawadee Osotprasit, Tepparit Samrit, Stuart J. Smith, Saowaros Suwansa-Ard, Scott F. Cummins, Pornanan Kueakhai, Narin Changklungmoa

**Affiliations:** 1 Faculty of Allied Health Sciences, Burapha University, Chonburi 20131, Thailand; 63810103@go.buu.ac.th (A.C.); 63810104@go.buu.ac.th (S.O.); tepparit.sa@go.buu.ac.th (T.S.); pornanan@go.buu.ac.th (P.K.); 2 Food Bioactive Compounds Research Unit, Faculty of Allied Health Sciences, Burapha University, Chonburi 20131, Thailand; 3 Centre for Bioinnovation, University of the Sunshine Coast, Maroochydore, QLD 4558, Australia; ssmith16@usc.edu.au (S.J.S.); ssuwansa@usc.edu.au (S.S.-A.); scummins@usc.edu.au (S.F.C.); 4 School of Science, Technology and Engineering, University of the Sunshine Coast, Sippy Downs, QLD 4558, Australia

**Keywords:** colon cancer, *Parkia speciosa*, anticancer activity, transcriptomics, proteomics

## Abstract

This study elucidates the multi-targeted antineoplastic mechanisms of *Parkia speciosa* empty pod extract (PSET) against HCT-116 and HT-29 colorectal cancer (CRC) cells through integrated transcriptomic and proteomic analyses. Phytochemical profiling indicates that PSET is rich in bioactive metabolites, notably quercetin, rutin, and pyrogallol, which orchestrate its profound ability to inhibit tumor proliferation, migration, and invasion. Transcriptomic data revealed that PSET profoundly suppresses the oncogenic Wnt/β-catenin signaling axis while simultaneously activating p53-mediated cell cycle arrest. Complementary proteomic profiling uncovered critical metabolic vulnerabilities, demonstrating that PSET abrogates the Warburg effect by disrupting key glycolytic enzymes (e.g., ENO1, GAPDH, LDHA), thereby inducing metabolic starvation. Furthermore, the extract precipitated a catastrophic collapse of the cytoskeletal architecture and downregulated epithelial–mesenchymal transition (EMT) markers, effectively paralyzing the cells’ metastatic machinery. The integrated transcriptomic and proteomic signatures also highlighted an irrecoverable state of cellular stress, characterized by an overwhelming unfolded protein response and dysregulated RNA splicing, ultimately driving the cells toward apoptosis. In conclusion, this integrated omics approach provides robust molecular validation that PSET systemically dismantles colorectal cancer survival networks, highlighting its strong potential as a natural, multi-targeted therapeutic agent.

## 1. Introduction

Colorectal cancer (CRC) remains a formidable challenge in global oncology, persistently ranking among the leading causes of cancer-related mortality worldwide [1]. The primary driver of this high lethality is not the localized tumor itself, but rather its metastatic dissemination—a highly complex, multi-step biological cascade that enables malignant cells to invade surrounding tissues and colonize distant organs [2]. While contemporary therapeutic modalities, including systemic chemotherapy and targeted biologics, have modestly improved patient survival rates, they are frequently constrained by severe, dose-limiting toxicities and the inevitable emergence of chemoresistance [3]. Consequently, there is an urgent, unmet clinical need to discover novel therapeutic agents that can effectively disrupt the metastatic cascade while preserving the functional integrity of non-malignant tissues [4]. Within this context, naturally derived bioactive compounds have garnered substantial attention as a promising, sustainable reservoir for the development of safer, next-generation antineoplastic drugs [5].

In the pursuit of novel phytochemical therapeutics, *Parkia speciosa* Hassk., a leguminous tree endemic to Southeast Asia, presents a compelling subject of investigation [6]. Traditionally celebrated for its culinary applications and ethnomedicinal properties, the seeds of *P. speciosa* have been extensively documented for their antioxidant, anti-inflammatory, and hypoglycemic benefits [6,7,8,9,10]. However, the agricultural harvesting and processing of this plant generate a massive volume of underutilized byproducts—specifically the empty pods—which are conventionally discarded as agro-industrial waste. Recent phytochemical profiling has fundamentally shifted this perspective, revealing that these discarded pods are, in fact, an abundant repository of potent secondary metabolites, particularly flavonoids and phenolic compounds [11,12,13,14,15]. The scientific valorization of these empty pods not only aligns with the modern principles of a circular bioeconomy but also offers a remarkably untapped resource for oncological drug discovery.

Building upon this premise, our preceding investigations sought to evaluate the anti-cancer potential of the ethanolic extract derived from *P. speciosa* empty pods (PSET) against human CRC. Recent in vitro investigations have underscored the previously untapped therapeutic potential of PSET in the field of oncology. Despite being historically discarded as agricultural waste, PSET has demonstrated a profound ability to combat human colorectal cancer by directly targeting the metastatic cascade. Specifically, the extract induces a significant, dose-dependent inhibition of colorectal cancer cell migration, invasion, and colony formation, all while remaining non-cytotoxic to normal colon epithelial cells. Mechanistically, this suppression is driven by the extract’s capacity to cripple the epithelial–mesenchymal transition (EMT) pathway, notably reducing the expression of critical extracellular matrix-degrading enzymes and adhesion molecules, including Matrix Metalloproteinases (MMP2 and MMP9), and N-cadherin [16]. These foundational findings highlight PSET as a promising, less toxic botanical candidate for halting colon cancer metastasis and provide a strong rationale for its development as an adjuvant therapeutic agent. Our foundational in vitro studies yielded highly encouraging results, demonstrating that PSET exerts a profound, dose-dependent inhibitory effect on the metastatic hallmarks of CRC cells, specifically curtailing their migratory, invasive, and clonogenic capabilities. Crucially, this aggressive anti-tumor activity was achieved without inducing significant cytotoxicity in normal colonic epithelial cells, indicating a highly favorable therapeutic index. At the cellular level, our initial data revealed that PSET effectively hindered the EMT—a pivotal biological reprogramming event hijacked by cancer cells to acquire motility—by downregulating the expression of critical prometastatic effectors, including MMP2, MMP9 and N-cadherin [16].

While these phenotypic observations and targeted protein analyses provided compelling evidence of PSET’s anti-metastatic efficacy, the precise intracellular mechanics driving these outcomes remain obscured. Cancer metastasis is not orchestrated by isolated pathways, but rather by an intricate, highly coordinated choreography of gene and protein networks [4]. Traditional reductionist approaches, which examine a handful of targets in isolation, are inherently insufficient to capture the global cellular reprogramming induced by PSET. To transition from phenomenological observations to a definitive mechanistic understanding, it is imperative to interrogate the cellular response at a systems biology level.

To bridge this critical knowledge gap, the current study is designed to elucidate the holistic molecular effects of PSET on CRC cells through a comprehensive integration of transcriptomic and proteomic analyses. By integrating advanced high-throughput transcriptomics with sophisticated proteomic profiling, we aim to map the complete landscape of gene expression alterations and protein interaction networks modulated by PSET treatment. This dual-omics strategy will allow us to decipher the complex signaling cascades, identify novel therapeutic targets, and delineate the exact molecular pathways responsible for its anti-tumor properties. Ultimately, the insights generated from this transcriptomic and proteomic perspective will not only validate the bioactivity of a discarded agricultural byproduct but also establish a robust, molecularly defined foundation for the clinical translation of PSET as an innovative, nature-derived intervention against metastatic CRC.

## 2. Results

### 2.1. Dose-Dependent Cell Toxicity of PSET Treatment

Previously, we determined the minimal dose of the PSET that could contrast the malignant phenotype of HCT-116 and HT-29 CRC cells with no cytotoxicity. All cell lines showed a dose-dependent reduction in cell viability by MTT assay [16]. The doses of PSET at 25–400 μg/mL did not induce cell death as early as 24 h-treatment, demonstrated by kept cell viability within the 95–105% range. Therefore, 300 μg/mL PSET was chosen for profiling via RNAseq and semi-quantitative proteomic analysis.

### 2.2. Transcriptomic Analysis of PSET Treatment

**Figure 1:**
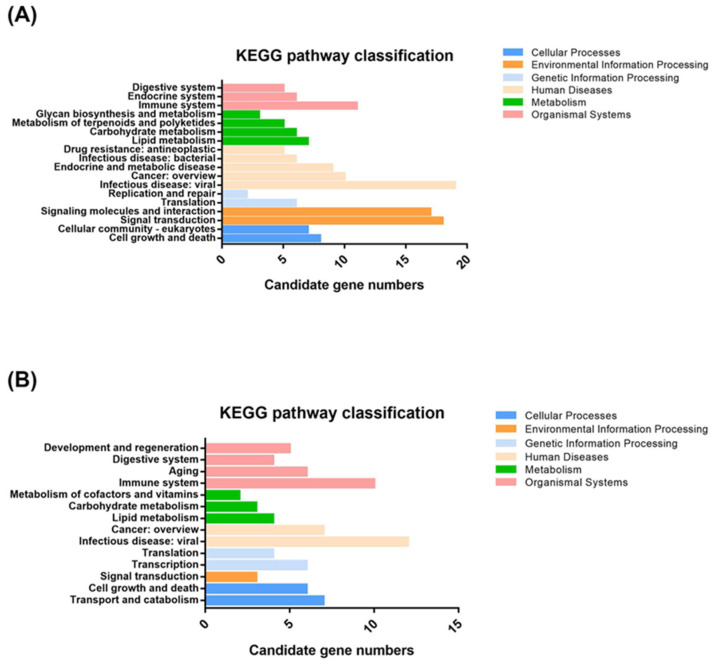
KEGG pathway classifications for the differentially expressed genes following PSET treatment. Classifications for genes in (**A**) HCT-116 cells and (**B**) HT-29 cells. The KEGG pathway classification provides an enriched string diagram with the corresponding relationship between the target gene and the KEGG pathway.

The effect of PSET treatment on gene expression in HCT-116 and HT-29 cells was profiled via RNAseq analysis. Differential RNAseq analysis was used to investigate the effect of PSET on colon cancer cells at the molecular level (Table 1). The results of the PSET treatment for HCT-116 cells revealed a total of 15,555 genes, of which 7662 were upregulated and 7893 were downregulated. For HT-29 cells, the results revealed a total of 15,366 genes, of which 7594 were upregulated and 7772 were downregulated compared to those in the untreated group. Of the significantly upregulated and downregulated differentially expressed genes (DEGs), 1631 genes (10.48%) were shared in the HCT-116 cell lines, while 1350 genes (8.78%) were shared in the HT-29 cell lines. The DEGs were identified and considered statistically significant at a *p* < 0.05. This statistical significance was determined using a paired t-test comparing the PSET-treated cells to the untreated control cells. Subsequent KEGG pathway analysis demonstrated that the majority were involved in metabolism, genetic information processing, human diseases, organismal systems and cellular processes (Figure 1). According to the KEGG pathways analysis, highly significant alteration was observed in the HIF-1, p53, NF-kappa B and NOD-like receptor signaling pathways.

**Figure 2:**
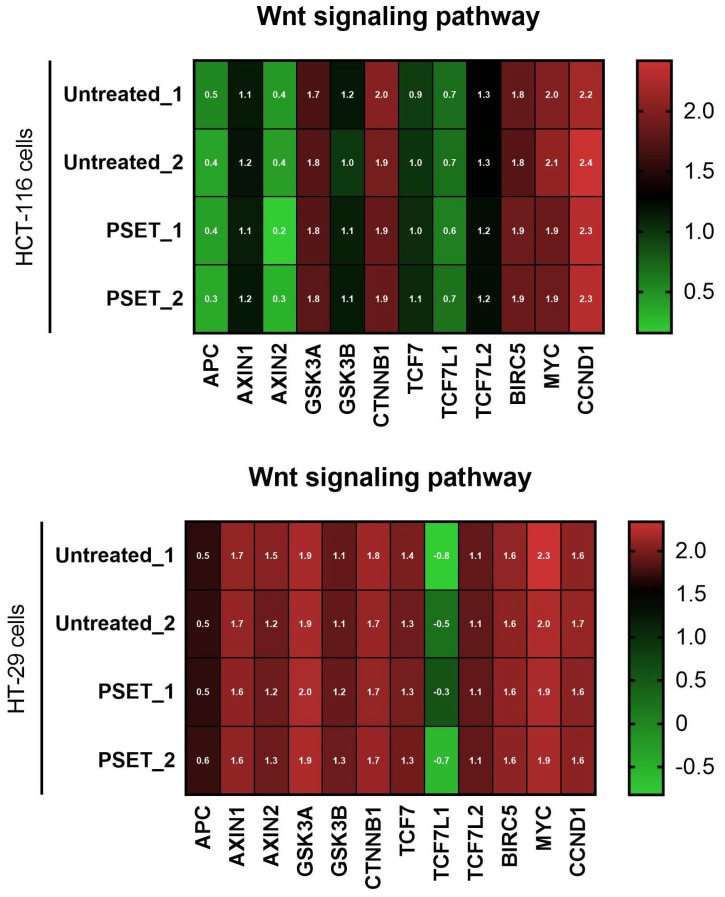
The heatmap for upstream gene (Wnt pathway) expression in HCT-116 and HT-29 cells. Data showed the log FPKM value compared to the control (Untreated cells).

**Figure 3:**
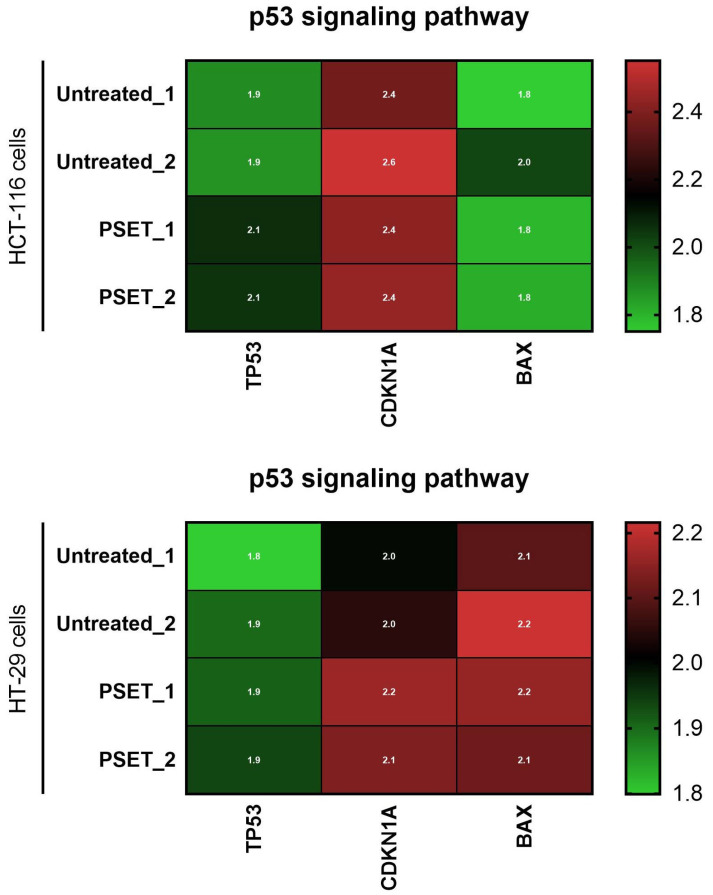
The heatmap for upstream gene (p53 pathway) expression in HCT-116 and HT-29 cells. Data showed the log FPKM value compared to the control (Untreated cells).

**Figure 4:**
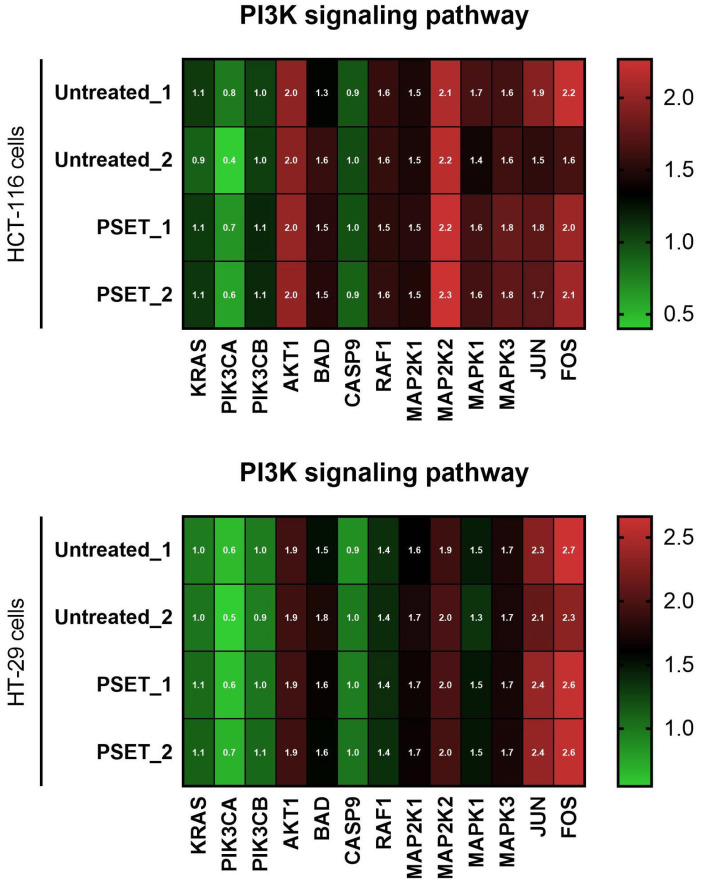
The heatmap for upstream gene (PI3K pathway) expression in HCT-116 and HT-29 cells. Data showed the log FPKM value compared to the control (Untreated cells).

**Figure 5:**
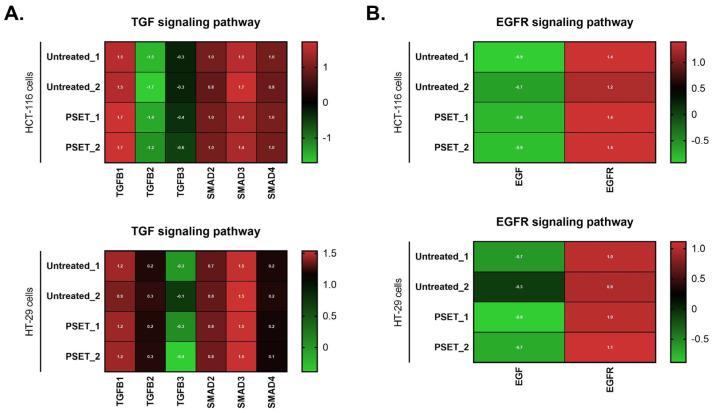
The heatmap for upstream gene (TGF (**A**) and EGFR (**B**) pathway) expression in HCT-116 and HT-29 cells. Data showed the log FPKM value compared to the control (Untreated cells).

Transcriptomic profiling demonstrated that PSET treatment significantly attenuated the expression of key components within the Wnt\β-catenin signaling cascade in both HCT-116 and HT-29 cell lines. Specifically, quantitative analysis revealed a marked downregulation of the central signal transducer CTNNB1. Concurrently, the transcript levels of its primary downstream proliferative targets, namely MYC and CCND1, exhibited a substantial reduction compared to the vehicle-treated controls (Figure 2). This antiproliferative profile was further reinforced by the distinct activation of the p53 tumor suppressor pathway; specifically, PSET treatment upregulated TP53 and its canonical downstream effector CDKN1A (p21) in both cell lines, with HT-29 cells exhibiting a particularly marked induction of CDKN1A, suggesting a cell-cycle arrest mechanism (Figure 3). However, the treatment response was not monolithic, as compensatory or stress-associated signaling was observed in parallel. Within the PI3K/MAPK network, PSET appeared to trigger adaptive feedback loops, characterized by the upregulation of MAP2K2 in HCT-116 cells and the profound activation of the AP-1 transcription factor JUN in HT-29 cells (Figure 4). Similarly, the TGF-β pathway displayed cell-type-specific uncoupling in HCT-116 cells, where increased TGFB1 ligand expression paradoxically coincided with SMAD3 effector suppression, whereas the EGFR pathway remained largely refractory to treatment across both cell lines, showing stable, low-level expression profiles irrespective of PSET exposure (Figure 5). Collectively, these data suggest that PSET’s therapeutic efficacy is likely driven by a dual mechanism of Wnt suppression and p53/p21-mediated cell cycle arrest, despite the concurrent activation of compensatory MAPK stress signals.

**Figure 6:**
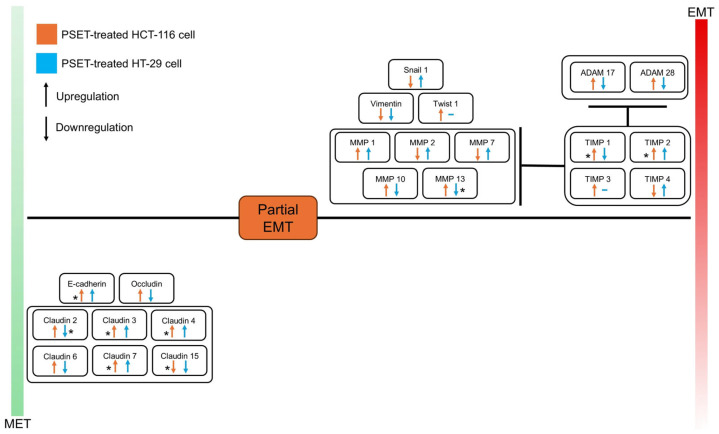
Downstream epithelial–mesenchymal transition (EMT) cancer pathways following PSET extract treatment of the HCT-116 and HT-29 cancer cell lines. The green and red bars represent the level and intensity of gene expression. Statistical analysis was performed using a paired *t*-test compared to untreated control cells. Asterisks indicate *p*-values: *, *p* < 0.05.

A detailed analysis of the expression data revealed that pathways related to cancer development were identified following the expression of the upstream pathway (Wnt, PI3K, TGF, p53 and EGFR). The results showed significant upregulation of AXIN2 gene in Wnt pathway and p53 gene and downregulation of SMAD3 gene in TGF pathway in HCT-116 cells compared with the untreated group (*p* < 0.05) (Table S1). Furthermore, the results for downstream of EMT and MET gene expression showed that the E-cadherin, claudin 3, claudin 4, claudin 7, TIMP 1 and TIMP 2 genes were upregulated in HCT-116 cells, while claudin 15 was downregulated in HCT-116 cells; claudin 3 and MMP 13 were downregulated in HT-29 cells, compared with the untreated group (*p* < 0.05) (Figure 6 and Table S1). Transcriptomic analyses showed that the inhibitory effects of the PSET on colon cancer cells may be associated with the potential modulations in upstream Wnt and p53 signaling and EMT-related pathways, since these pathways were highly enriched with significantly altered genes.

### 2.3. Proteomic Analysis of PSET Treatment

**Figure 7:**
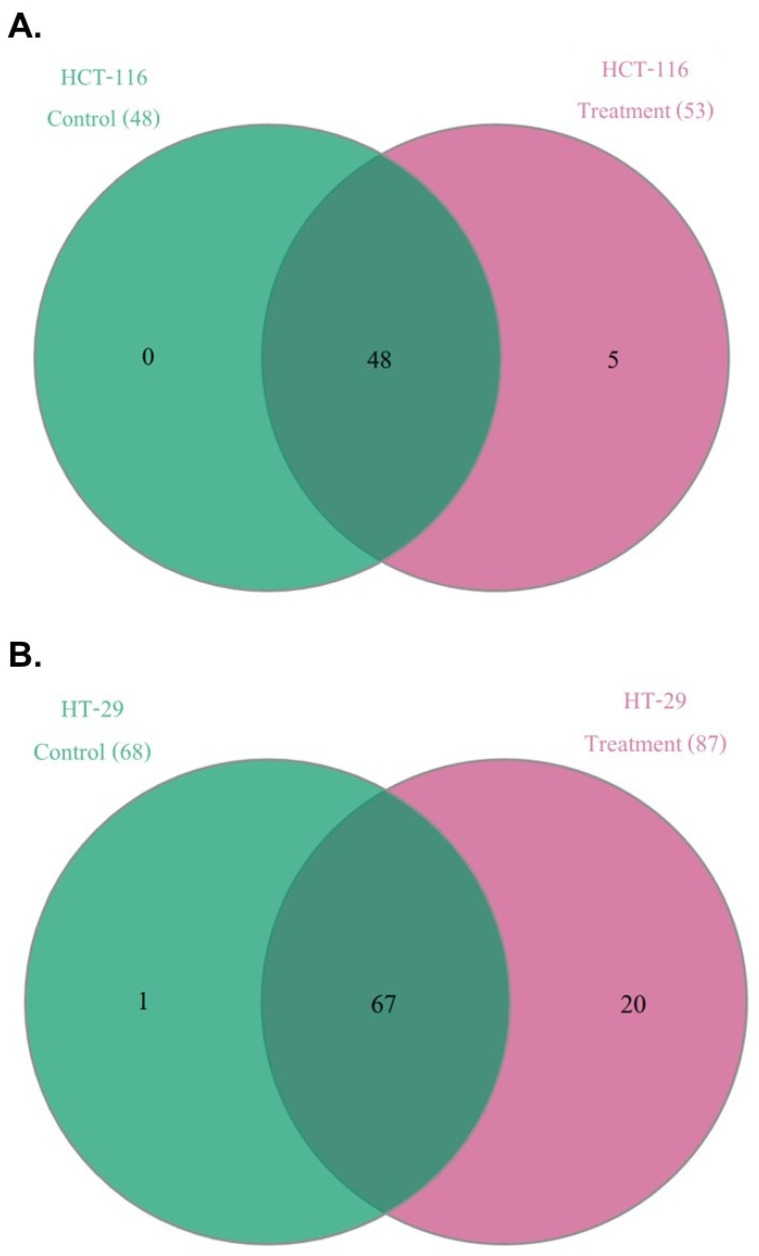
Profiling of altered protein groups in HCT-116 cells (**A**) and HT-29 cells (**B**).

**Figure 8:**
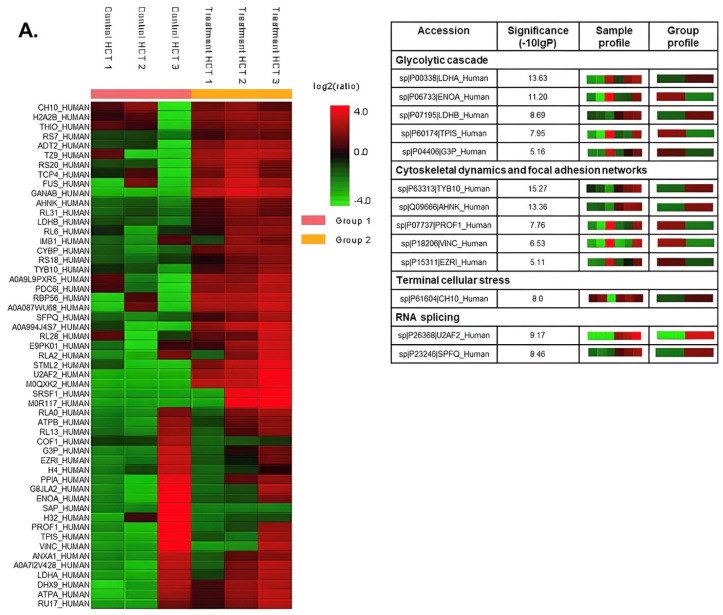

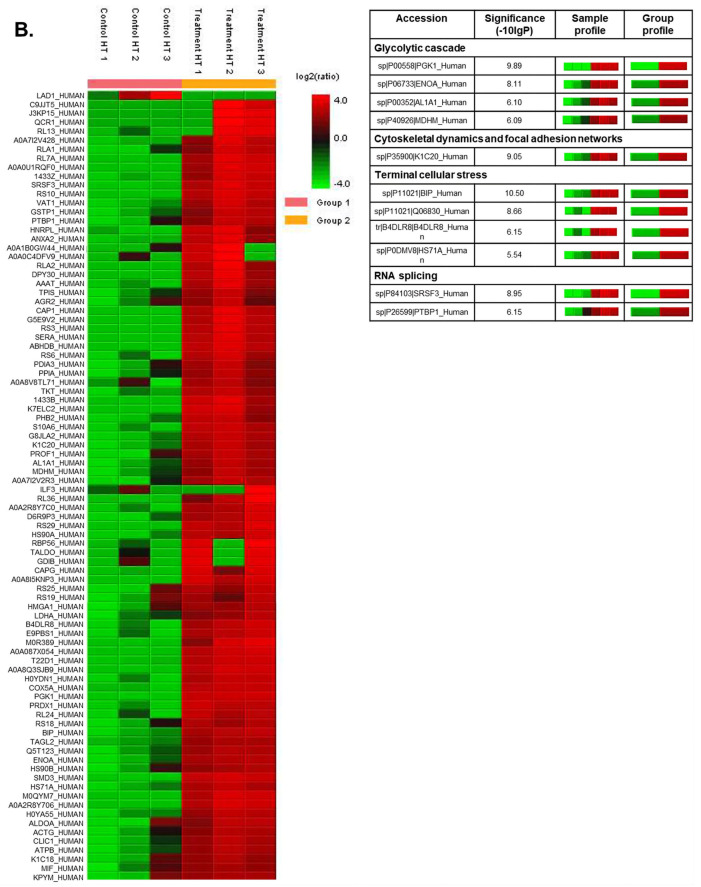
Proteomic changes caused by the treatment with PSET. Untargeted proteomic analysis of HCT-116 and HT-29 cells after treatment with 300 µg/mL PSET. Heat maps illustrate the relative abundance of differentially expressed proteins in treated HCT-116 (**A**), and HT-29 (**B**) cells compared to their respective controls. Each group contained three biological replicates.

To evaluate the global protein expression alterations induced by PSET, a semi-quantitative label-free proteomic analysis was performed on HCT-116 (Table S2) and HT-29 (Table S3) CRC cell lines. Quantitative analysis, represented in the Venn Diagrams (Figure 7), identified 53 significantly differentially expressed protein groups in HCT-116 cells and 87 in HT-29 cells, based on stringent statistical thresholds (FDR < 0.05, Fold change ≥ 1.2). Hierarchical clustering analysis demonstrated distinct protein expression profiles that clearly separated the PSET-treated cohorts from the untreated controls (Figure 8). Functional categorization of these differentially expressed proteins indicated their primary involvement in cellular metabolism, cytoskeletal organization, and stress response alongside RNA processing.

The proteomic data revealed the significant differential expression of several key enzymes associated with cellular energy metabolism and the glycolytic cascade. Specifically, PSET treatment resulted in the altered expression of LDHA, LDHB, TPI1, and GAPDH in HCT-116 cells, as well as PGK1, ALDH1A1, and MDH2 in HT-29 cells (Figure 8). Furthermore, the expression of alpha-enolase (ENO1) exhibited a lineage-dependent differential response following treatment. In the wild-type p53 HCT-116 cells, ENO1 expression was significantly downregulated. Conversely, in the mutant p53 HT-29 cell line, the expression levels of ENO1 were found to be elevated.

In addition to metabolic enzymes, the proteomic profiles indicated significant changes in the abundance of proteins that constitute the cytoskeletal architecture and focal adhesion networks. In HCT-116 cells, PSET treatment led to the significant dysregulation of several structural and actin-binding proteins, including PFN1 (Profilin-1), TMSB10, EZR, VCL, and AHNAK. Concurrently, significant alterations in the expression of the intermediate filament protein KRT20 were detected within the treated HT-29 cells (Figure 8).

Finally, the proteomic analysis detected the upregulation of key molecular chaperones and stress-response proteins in both cell lines following PSET exposure. HSPE1 expression was significantly upregulated in HCT-116 cells, whereas HSPA5 (BIP), HSPA1A, PRDX1, and NQO1 exhibited pronounced upregulation in HT-29 cells (Figure 8). Alongside these stress-response markers, proteins involved in the RNA processing apparatus exhibited significantly altered expression profiles. Essential RNA splicing factors, including SFPQ and U2AF2 in HCT-116 cells, as well as SRSF3 and PTBP1 in HT-29 cells, were found to be significantly dysregulated following PSET intervention.

## 3. Discussion

The integrated transcriptomic and proteomic profiling of HCT-116 and HT-29 CRC cell lines elucidates the multi-targeted antineoplastic mechanisms of PSET. The molecular reprogramming revealed by transcriptomic and proteomic approaches provides a comprehensive mechanistic foundation that aligns seamlessly with previous in vitro functional studies, which demonstrated PSET’s profound capacity to inhibit CRC cell migration, invasion, and colony formation in a dose-dependent manner [16]. Crucially, advanced chemical profiling via LC-MS/MS and GC-MS has previously revealed that PSET is a rich reservoir of bioactive secondary metabolites, including saponins, alkaloids, terpenoids, and polyphenols [15]. The extract is replete with prominent phytochemicals such as pyrogallol (1,2,3-benzenetriol), quercetin, rutin, gallic acid, epigallocatechin, theasinensin A, myricitrin, eucommin A, palmitoleic acid, vitamin E, and alpha-amyrin [15]. The synergistic interplay of these constituents provides a robust mechanistic rationale for the molecular alterations observed in our data.

Previous phenotypic evaluations established that PSET significantly suppresses CRC cell viability and severely cripples their reproductive potential [16]. The in vitro efficacy of PSET presents a sophisticated anti-cancer profile that notably uncouples generalized cytotoxicity from targeted anti-metastatic action. While high concentrations (exceeding 600 µg/mL) are required to induce significant cell death, PSET functions as a highly effective “molecular brake” at sub-lethal concentrations ranging from 50 to 200 µg/mL. At these lower doses, the extract actively paralyzes the invasive capabilities of HT-29 and HCT-116 cells without compromising overall cell viability. This targeted intervention is molecularly validated by the robust downregulation of MMP2, MMP9, and N-cadherin, indicating that PSET actively reverses the invasive mesenchymal programming of tumor cells, effectively locking them in a non-invasive state [16]. The potent, multi-target efficacy observed is likely driven by the synergistic actions of PSET’s abundant phytochemical constituents—such as pyrogallol, quercetin, and rutin—which collectively dismantle the metastatic machinery and inhibit extracellular matrix degradation. Pharmacologically, this discrepancy between cytotoxic and anti-metastatic thresholds represents a highly desirable therapeutic profile. Consequently, PSET offers a compelling foundation for the development of safer, targeted therapies designed to prevent colorectal cancer dissemination without exacerbating the severe systemic toxicity commonly associated with conventional high-dose chemotherapeutics.

The transcriptomic landscape elucidates that PSET treatment orchestrates a complex, multi-targeted reprogramming of cellular signaling, primarily functioning as a potent inhibitor of the canonical Wnt axis while simultaneously engaging stress-responsive and tumor-suppressive mechanisms. Our transcriptomic data elucidate the genetic basis for this anti-proliferative effect, revealing that PSET profoundly suppresses the canonical Wnt/β-catenin signaling axis. The targeted downregulation of the core transducer CTNNB1 and its downstream oncogenic effectors, MYC and CCND1 (Cyclin D1), effectively removes the intrinsic proliferative drive. Concomitantly, PSET upregulates the TP53 (p53) tumor suppressor gene and its downstream kinase inhibitor CDKN1A (p21), driving the cells into definitive cell cycle arrest. These molecular alterations are highly consistent with the known mechanisms of the predominant flavonoids found in PSET. Specifically, quercetin is well-documented to modulate the Wnt/β-catenin pathway, arrest the cell cycle in the G2/M phase, and induce apoptosis [17,18,19,20]. Furthermore, pyrogallol acts as a potent cytostatic agent that arrests the cell cycle and dismantles the oncogenic RAS/PI3K/AKT/mTOR signaling axis [21,22,23,24,25,26].

The lethal progression of CRC is largely driven by metastasis, a highly demanding cellular journey dependent on motility and extracellular matrix (ECM) degradation [27]. Prior findings confirmed that PSET paralyzes the metastatic machinery of tumor cells, significantly inhibiting migration and invasion by downregulating key markers of the EMT, notably MMP2, MMP9, and N-cadherin [16]. Our proteomic analysis corroborates and expands upon these findings by demonstrating a catastrophic collapse of the internal cytoskeletal architecture. PSET induced marked dysregulation of crucial structural and actin-binding proteins, including PFN1, TMSB10, EZR, VCL, and KRT20. This dismantling of cellular structural integrity perfectly mirrors the “double-hit” pharmacological strategy exerted by PSET’s phytochemicals. Quercetin directly targets the molecular machinery of metastasis by inhibiting the expression of MMP2 and MMP9, thereby preventing physical invasion and locking cells in a non-invasive state. Simultaneously, rutin impairs cell adhesion and mitigates migration [28,29,30,31]. Together, these bioactives function as a structural “molecular brake” that synergizes with the suppression of proteolytic enzymes to comprehensively halt mesenchymal drift.

A critical novel insight provided by our proteomic data is the identification of severe metabolic vulnerability induced by PSET. The treatment precipitated a profound disruption of the glycolytic infrastructure, characterized by the significant dysregulation of pivotal metabolic enzymes such as ENO1, GAPDH, PGK1, and LDHA [32,33]. This widespread interference effectively abrogates the Warburg effect, depriving the malignant cells of the rapid ATP generation required to fuel highly energy-consuming processes like ECM invasion and uncontrolled proliferation [34,35,36]. The diverse array of phenolic compounds in PSET, such as epigallocatechin and gallic acid, likely contributes to this profound disruption of cellular energy homeostasis. The induction of this metabolic starvation state serves as a critical upstream mechanism that further explains the extract’s ability to impair colony formation and halt the physically demanding processes required for metastasis.

By explicitly integrating our transcriptomics and proteomics data, we demonstrate that the transcriptomic and proteomic datasets converge to tell a single, cohesive biological story detailing how PSET systemically dismantles colorectal cancer survival networks. This combined analysis reveals a distinct mechanistic flow where upstream genetic reprogramming dictates downstream functional and metabolic execution; specifically, transcriptomic profiling indicates that PSET profoundly suppresses the oncogenic Wnt/β-catenin signaling axis through the targeted downregulation of CTNNB1, MYC, and CCND1, while simultaneously driving cell cycle arrest via the activation of TP53 and CDKN1A. Corroborating and functionally expanding upon these genetic alterations, our complementary proteomic data uncovers a catastrophic collapse of the internal cytoskeletal architecture—marked by the dysregulation of structural proteins such as PFN1, TMSB10, EZR, VCL, and KRT20—which, alongside the downregulation of epithelial–mesenchymal transition markers, effectively paralyzes the cells’ metastatic machinery. Furthermore, this proteomic perspective highlights severe downstream metabolic vulnerabilities, demonstrating that PSET abrogates the Warburg effect by disrupting key glycolytic enzymes like ENO1, GAPDH, and LDHA, thereby inducing a state of metabolic starvation. Ultimately, the convergence of these omics layers illustrates that the upstream transcriptomic induction of tumor suppression, paired with the proteomic reality of structural dismantling, extreme cellular stress, and an overwhelming unfolded protein response, synergistically seals the apoptotic fate of the malignant cells.

Finally, the transcriptomics and proteomics signatures highlight the induction of an irrecoverable state of cellular stress. Proteomic profiling revealed the massive upregulation of critical molecular chaperones and stress-response proteins, such as HSPA5 (BIP), HSPA1A, and PRDX1, alongside widespread dysregulation of the RNA splicing machinery. This indicates an overwhelming unfolded protein response (UPR) and severe mitochondrial distress [37,38]. This molecular evidence of critical cellular stress strongly corroborates the pharmacological profiles of PSET’s constituents. Rutin, while typically an antioxidant, can paradoxically increase reactive oxygen species (ROS) generation to toxic levels within malignant tissues, leading to lethal DNA damage [39]. Similarly, pyrogallol is documented to inflict targeted DNA damage upon malignant cells and trigger apoptotic cell death [40]. The accumulation of this severe intracellular damage, combined with targeted metabolic starvation and structural collapse, ultimately seals the apoptotic fate of the cancer cells.

## 4. Materials and Methods

### 4.1. Plant Extraction

The present study utilized a pre-characterized sample batch of *P. speciosa* empty pods (lot: PS-P-2023-001). The phytochemical profiling has been delineated by Samrit et al. [15]. Utilizing Liquid Chromatography–Tandem Mass Spectrometry (LC-MS/MS) coupled with Gas Chromatography–Mass Spectrometry (GC-MS), their comprehensive profiling established the abundant presence of key bioactive secondary metabolites, most prominently the potent phenolic and flavonoid compounds pyrogallol, rutin, and quercetin. The resulting crude extract was dissolved in dimethyl sulfoxide (DMSO; Sigma-Aldrich, Saint Louis, MO, USA), thereby generating a standardized master stock solution at a concentration of 1 mg/mL. Consequently, the final working concentration of the DMSO vehicle was strictly maintained below a critical physiological threshold of 1% (*v*/*v*). A matched vehicle control group receiving the equivalent concentration of DMSO (<1% *v*/*v*) was included in all transcriptomic and proteomic experiments to ensure observed effects were specific to the PSET.

### 4.2. Cell Cultures

All cell lines were purchased from the American Type Culture Collection (ATCC). Specifically, the non-tumorigenic human colon fibroblast CCD-18Co (ATCC No. CRL-1459™; Lot No. 70024131) was resuscitated and initiated at passage 11. To represent diverse molecular landscapes of CRC malignancy, the human colon carcinoma cell line HCT-116 (ATCC No. CCL-247™; Lot No. 70019042) was established at passage 6 post-receipt, while the human colon adenocarcinoma cell line HT-29 (ATCC No. HTB-38™; Lot No. 70035209) was cultured beginning at passage 129. CCD-18Co was propagated in Dulbecco’s Modified Eagle Medium (DMEM), explicitly supplemented with 1 g/L D-glucose, L-glutamine, and 110 mg/L sodium pyruvate. Conversely, HCT-116 and HT-29 were sustained in McCoy’s 5A modified medium, formulated with L-glutamine without sodium bicarbonate. To completely preclude microbial contamination while providing indispensable exogenous growth factors, both basal media formulations were supplemented with 10 U/mL penicillin G and 10 μg/mL streptomycin alongside 10% (*v*/*v*) fetal bovine serum (FBS). All cell lines were cultivated at 37 °C in a humidified incubator with a 5% CO_2_ atmosphere and monitored daily. The culture medium was replenished at 48- to 72-h intervals. Cells at the logarithmic (exponential) growth phase were harvested before performing each experiment. For all experiments, passage numbers of CCD-18Co (passages 14 to 17), HT-29 (passages 133 to 136) and HCT-116 (passaged ten to thirteen times after receipt) were used to ensure phenotypic stability.

### 4.3. RNA Extraction and Transcriptomic Analysis

HCT-116 and HT-29 cells were seeded in 6-well plates at a density of 1 × 10^6^ cells per well and cultured for 24 h at 37 °C in a humidified 5% CO_2_ atmosphere. Then, the cells were treated with PSET at concentrations of 300 µg/mL for 24 h. After treatment, the cells were harvested by scraping and centrifugation at 1000× *g* for 5 min. The cell pellets obtained were washed with PBS and stored at −80 °C until RNA extraction.

Total RNA was isolated via TRIzol™ Reagent (Thermo Fisher Scientific, Waltham, MA, USA). Following the homogenization of cell pellets in 0.75 mL of the reagent and the addition of 0.2 mL chloroform. After 2 min room-temperature incubation, phase separation was achieved via centrifugation at 12,000× *g* for 15 min at 4 °C. The RNA-enriched upper aqueous fraction was transferred into a new tube, and nucleic acid precipitation was catalyzed using 0.5 mL of isopropanol with a 10 min incubation at 4 °C. Following a secondary sedimentation step (12,000× *g*, 10 min, 4 °C), the resulting RNA precipitate was desalted using 1 mL of 75% ethanol. The purified pellet was then air-dried to remove residual solvent and reconstituted in 50 µL of RNase-free water supplemented with 0.1 mM EDTA. To ensure complete solubilization, the final suspension was subjected to incubate at 55 °C for 10 min prior to long-term cryopreservation at −80 °C.

RNA-sequencing was performed using two independent biological replicates per experimental group (Untreated and PSET-treated). While traditional standalone RNA-seq analyses typically employ a minimum of three biological replicates, the transcriptomic profiling in this study was specifically designed as a system-level screening tool to identify global shifts in signaling cascades. To ensure the reliability and statistical robustness of our molecular findings, the RNA-seq dataset was integrated within a comprehensive transcriptomic and proteomic framework. All critical pathway alterations identified via transcriptomics—including the modulation of the Wnt/β-catenin, p53, PI3K, TGF, and EGFR signaling axes—were subsequently cross-validated using semi-quantitative proteomics. All proteomic analyses and subsequent in vitro functional assays were independently conducted with three biological replicates (*n* = 3) to establish rigorous biological validity.

RNA sequencing and library preparation were executed by an external genomics facility (BGI Hong Kong Tech Solution NGS Lab, Tai Po, Hong Kong; Project ID: F23A430002074_HOMkcpqR). Library construction commenced with the targeted enrichment of polyadenylated mRNA from the total RNA isolates, utilizing oligo(dT)-functionalized magnetic beads. Following RNA fragmentation, reverse transcription was performed to generate complementary DNA. The resulting double-stranded cDNA constructs were subsequently purified, enriched via PCR amplification, and subjected to high-throughput sequencing utilizing the BGIseq-500 platform. For downstream in silico analysis, raw sequencing reads were aligned to the NCBI *Homo sapiens* reference genome (Assembly: GCF_000001405.39_GRCh38.p13). Transcript abundance was normalized using the Fragments Per Kilobase of transcript per Million mapped reads (FPKM) metric. Subsequent functional annotations, KEGG pathway enrichment analyses, were performed leveraging BGI’s proprietary Dr. TOM bioinformatics data-mining platform. All raw sequencing data generated during this study have been deposited in the NCBI Sequence Read Archive (SRA) and are publicly accessible under the accession number PRJNA1451310.

### 4.4. Sample Preparation and Protein Digestion

HCT-116 and HT-29 were plated into 6-well plates at a seeding density of 1 × 10^6^ cells per well. Cultures were maintained in complete McCoy’s 5A medium and allowed to adhere for 24 h under standard physiological conditions (37 °C in a humidified 5% CO_2_ atmosphere). Following the initial equilibration phase, the cells were subjected to experimental treatment with PSET at concentrations of 300 µg/mL and incubated for an additional 24 h. Post-treatment, cells were harvested via scraping, followed by centrifugation at 1000× *g* for 5 min at room temperature. The resulting pellets were washed with phosphate-buffered saline (PBS). Total cellular protein was then extracted by resuspending the pellets in 600 µL of a lysis buffer comprising 8 M urea, 0.8 M ammonium bicarbonate (NH_4_HCO_3_, pH 8.0), and the protease inhibitor PMSF. Lysates were sonicated on ice for 2 min at 40% amplitude with 10 s on/off cycles and clarified by centrifugation at 12,000× *g* for 15 min at 4 °C. Supernatants were recovered, and protein concentrations were determined using the Pierce BCA protein assay.

For protein digestion, 100 µg of total protein was first subjected to 5 µL of 100 mM dithiothreitol (DTT) with a 1 h incubation at 37 °C, followed by 20 µL of 100 mM iodoacetamide (IAA) for 1 h at room temperature, strictly protected from light. The samples were then further incubated with 20 µL of 200 mM DTT for 1 h at room temperature. The urea concentration was lowered by diluting the samples with 775 µL of Milli-Q water. Enzymatic digestion was then catalyzed by the addition of sequencing-grade modified trypsin. The proteolytic reaction was allowed to proceed overnight at 37 °C under continuous, gentle agitation (20 rpm) to maximize peptide yield. Following overnight incubation, the tryptic digestion was terminated by acidifying the sample to a pH below 3.0, utilizing 10% formic acid (FA). Tryptic peptides were subjected to solid-phase extraction utilizing Sep-Pak C18 cartridges to desalt the sample and eliminate hydrophilic contaminants. The purified tryptic peptides were subsequently lyophilized to complete dryness in a vacuum concentrator (SpeedVac) and finally reconstituted in 50 µL of 0.1% FA for liquid chromatography-mass spectrometry (LC-MS) analysis.

15 µL of the peptide samples were injected into an Agilent AdvanceBio Peptide Map column (2.1 × 150 mm, 2.7 µm particle size) interfaced with an Exion LC™ system (AB SCIEX, Marlborough, MA, USA). Peptide fractionation was achieved utilizing a reverse-phase binary solvent architecture comprising 0.1% formic acid in water (Mobile Phase A) and 0.1% formic acid in acetonitrile (Mobile Phase B). Elution was executed over an optimized 52-min gradient. The gradient elution initiated with a 5-min span of 100% Mobile Phase A to desalt the loaded sample. This was followed by linear decrease to 60% Mobile Phase A for over 40 min, and then to 5% of solvent A over 5 min, concluding with a 2-min column re-equilibration at initial conditions (100% Mobile Phase A). Blank runs were executed at the beginning and end of each sample batch, while wash sequences were inserted between the individual samples. Eluting peptides were introduced into the mass spectrometer via an electrospray ionization (ESI) source operating in positive ion mode. The source parameters were tuned to maximize peptide volatilization and ion transmission: the capillary spray voltage was set to 5500 V, alongside a declustering potential (DP) of 100 V, a curtain gas flow of 30 psi, a nebulizer gas (GS1) setting of 12, and an interface heater maintained at 450 °C. Data acquisition utilized a time-of-flight (TOF) mass analyzer. A full TOF-MS survey scan was continuously acquired over a mass-to-charge (*m/z*) range of 350–1800 with a cycle time of 1.62 s. For downstream structural elucidation and peptide sequencing, precursor ions were dynamically selected for fragmentation based on stringent criteria: only charged species (charge states +2 to +5) demonstrating an intensity threshold exceeding 100 counts were isolated. The resulting product ion MS/MS spectra were subsequently recorded across an *m*/*z* range of 50 to 1800.

### 4.5. Protein Identification and Functional Annotation

Raw mass spectrometric data were processed utilizing PEAKS Studio software (Version 7.0; Bioinformatics Solutions Inc., Toronto, ON, Canada). To maximize proteome coverage and peptide identification confidence, the analytical pipeline integrated de novo sequencing algorithms with traditional database searching and automated post-translational modification (PTM) discovery. Acquired MS/MS spectra were interrogated against a comprehensive *Homo sapiens* reference proteome (UniProt assembly UP000005640, comprising 82,485 discrete protein sequences). To ensure rigorous statistical stringency, peptide-spectrum matches were tightly constrained by calculating the target-decoy threshold score [−10xlog(p)], restricting the global false discovery rate (FDR) to a maximum of 0.5%. High-resolution database search parameters utilized monoisotopic mass values exclusively. The precursor ion mass tolerance was strictly confined to 5 ppm, while fragment ion mass tolerance was set to 0.1 Da. In silico proteolytic constraints mirrored the experimental trypsinization, permitting a maximum of three missed cleavage events per peptide sequence. To accurately capture the complex structural landscape of the sample, dynamic computational searching incorporated both static and variable modifications. Carbamidomethylation of cysteine residues, resulting from the alkylation protocol, was universally assigned as a fixed modification. Conversely, the algorithm accommodated a suite of variable modifications to detect dynamic biological and artifactual alterations: N-terminal pyroglutamate formation (originating from glutamine and glutamic acid), oxidation of methionine, deamidation of asparagine and glutamine, and acetylation of lysine. Adhering to open-science mandates, the complete, unedited mass spectrometry proteomics dataset has been formally deposited into the ProteomeXchange Consortium infrastructure. The mass spectrometry proteomics data have been deposited to the ProteomeXchange Consortium via the PRIDE [41] partner repository with the dataset identifier PXD078012 and 10.6019/PXD078012.

## 5. Conclusions

The integration of transcriptomic, proteomic, and phytochemical analyses provides robust molecular validation of PSET’s efficacy against CRC. By delivering a complex cocktail of bioactive flavonoids and phenolic compounds—most notably quercetin, rutin, and pyrogallol—PSET acts as a highly potent, multi-targeted antineoplastic agent. It systematically enforces p53-mediated cell cycle arrest, dismantles the cytoskeletal and EMT-associated metastatic machinery, induces metabolic starvation, and exacerbates lethal cellular stress. These comprehensive findings strongly support the continued development of PSET as a natural, biodegradable, and less toxic therapeutic alternative or dietary supplement for the management of CRC. A notable limitation of the present study is the use of only two biological replicates for RNA-sequencing and the lack of independent validation of selected gene and protein expression levels (e.g., RT-qPCR or Western blotting), which may reduce the statistical power of the transcriptomic analysis and warrant further confirmation of specific molecular targets. To firmly mitigate this limitation, we deliberately employed a transcriptomic and proteomic cross-validation strategy. The transcriptomic data was utilized primarily to guide and inform our broader investigation, rather than serving as standalone evidence. The high degree of concordance between our transcriptomic screening, our semi-quantitative proteomic data (*n* = 3), and our functional in vitro phenotypic assays (*n* = 3) provides robust confidence in the identified molecular mechanisms. Future studies focusing exclusively on the targeted transcriptional regulation of these pathways will benefit from expanded RNA-seq cohorts.

## Figures and Tables

**Table 1:** Summary of RNAseq analysis following cell line treatment with PSET.

Cell Line (Concentration)	Total DEGs	Total Upregulated	Total Downregulated
HCT-116 (300 µg/mL extract)	15,555	7662	7893
HT-29 (300 µg/mL extract)	15,366	7594	7772

## Data Availability

The datasets generated and analyzed during the current study are publicly available. The raw data from whole transcriptome sequencing are deposited under the BioProject accession number PRJNA1451310 in both the NCBI BioProject and SRA databases, accessible at https://www.ncbi.nlm.nih.gov/bioproject/PRJNA1451310 (accessed on 9 April 2026). Raw data from peptide mass spectrometry are accessible under the accession number PXD078012 at https://www.ebi.ac.uk/pride/archive/projects/PXD078012 in the PRIDE database (accessed on 5 May 2026).

## Supplementary Materials

The following supporting information can be downloaded at: https://www.mdpi.com/article/10.3390/ijms27125606/s1.

## Abbreviations

The following abbreviations are used in this manuscript:

DEGs	Differentially expressed genes
DP	Decluttering potential
DTT	Dithiothreitol
FA	Formic acid
GC-MS	Gas chromatography–mass spectrometry
GO	Gene Ontology
IAA	Alkylating reagent
LC-MS	Liquid Chromatography–Mass Spectrometry
NIST	National Institute of Standards and Technology
RNAseq	RNA sequencing
PMSF	Phenyl-Methyl-Sulfonyl-Fluoride
QTOF	Quadrupole Time-of-Flight
SRA	Sequence Read Archive
